# Association of hemodynamic factors and progressive aortic dilatation following type A aortic dissection surgical repair

**DOI:** 10.1038/s41598-021-91079-5

**Published:** 2021-06-01

**Authors:** Yu Zhu, Saeed Mirsadraee, George Asimakopoulos, Alessia Gambaro, Ulrich Rosendahl, John Pepper, Xiao Yun Xu

**Affiliations:** 1 Department of Chemical Engineering, Imperial College London, London, SW7 2AZ UK; 2 Department of Radiology, Royal Brompton and Harefield Hospitals NHS Trust, London, SW3 6NP UK; 3 Department of Cardiac Surgery, Royal Brompton and Harefield Hospitals NHS Trust, London, SW3 6NP UK; 4 Department of Cardiology, Royal Brompton and Harefield Hospitals NHS Trust, London, SW3 6NP UK

**Keywords:** Cardiology, Diseases, Engineering, Mathematics and computing

## Abstract

Type A aortic dissection (TAAD) involves the ascending aorta or the arch. Acute TAAD usually requires urgent replacement of the ascending aorta. However, a subset of these patients develops aortic rupture due to further dilatation of the residual dissected aorta. There is currently no reliable means to predict the risk of dilatation following TAAD repair. In this study, we performed a comprehensive morphological and hemodynamic analysis for patients with and without progressive aortic dilatation following surgical replacement of the ascending aorta. Patient-specific models of repaired TAAD were reconstructed from post-surgery computed tomography images for detailed computational fluid dynamic analysis. Geometric and hemodynamic parameters were evaluated and compared between patients with stable aortic diameters (N = 9) and those with aortic dilatation (N = 8). Our results showed that the number of re-entry tears and true/false lumen pressure difference were significantly different between the two groups. Patients with progressive aortic dilatation had higher luminal pressure difference (6.7 [4.6, 10.9] vs. 0.9 [0.5, 2.3] mmHg; P = 0.001) and fewer re-entry tears (1.5 [1, 2.8] vs. 5 [3.3, 7.5]; P = 0.02) compared to patients with stable aortic diameters, suggesting that these factors may serve as potential predictors of aneurysmal dilatation following surgical repair of TAAD.

## Introduction

Aortic dissection starts with a tear in the intima of the thoracic aorta, allowing blood to enter the medial layer to create a double lumen aorta. A dissected aorta usually consists of a true lumen (TL) and a false lumen (FL) which are separated by an intimal flap. The Stanford classification system divides aortic dissection into type A and B, depending on the location of the initial intimal tear. Type A dissection involves the ascending aorta and/or the arch, while type B dissection only involves the descending aorta. The standard surgical approach for Stanford type A aortic dissection (TAAD) repair is to replace the ascending aorta with a synthetic graft. Following repair, 12% of deaths were reported to be caused by aortic rupture due to further dilatation of the residual dissected aorta^1^. Aortic dilatation occurs in 49% of patients with a patent FL after repair of acute TAAD with onset from 1 to 167 months^2^.

Surgical repair of TAAD with replacement of the ascending aortic segment often leaves re-entry tears in the arch and descending aorta unattended, which may lead to late progressive aortic dilatation^3^. Patients with residual aortic dissection that have increased risk of developing aneurysmal dilatation would benefit from early interventions to prevent sudden aortic rupture and late death. However, there is currently no reliable means to predict the risk of dilatation following TAAD repair.

Several anatomical studies of type B aortic dissection (TBAD) have reported potential associations between anatomical features of the dissected aorta and late adverse outcomes. These include FL patency, larger aortic diameter, FL area, as well as the size and location of the entry tears^4–9^. In addition to anatomical features, hemodynamic characteristics in TBAD have been examined by applying computational fluid dynamics (CFD) to patient-specific geometry reconstructed from medical images^10–13^. Moreover, the influence of morphological variations on hemodynamic changes in TBAD has been evaluated^14–18^. It was suggested that hemodynamic indices derived from CFD could potentially predict FL growth and aneurysmal degeneration in TBAD^19–22^. Previous studies are promising, but were limited to TBAD. A very recent study using 4D flow magnetic resonance imaging demonstrated global and regional hemodynamic differences between TBAD and repaired TAAD^23^, confirming the need for further understanding of flow in surgically repaired TAAD.

In this study, we evaluated anatomical and hemodynamic features of different groups of surgically repaired TAAD. Based on post-surgery computed tomography angiography (CTA) images, patient-specific models were reconstructed and divided into two groups: patients with stable aortic diameters (N = 9) and those with progressive aortic dilatation (N = 8). Anatomical parameters were measured and compared between the two groups; these included primary entry tear size and location, number of re-entry tears and distance between tears, maximum aortic diameter, curvature ratio and tortuosity of the aorta, as well as true and false lumen volume ratio. Furthermore, patient-specific CFD simulations were performed and hemodynamic results including flow patterns, wall shear stress and pressures were compared. The aim of this study was to understand if there are any significant anatomical and hemodynamic differences between the two groups, and whether any of these factors might be potentially predictive of progressive aortic dilatation in surgically repaired TAAD.

## Results

This retrospective study was based on the validated database of patients with repaired TAAD at the Royal Brompton and Harefield hospitals. All patients were initially treated by replacing the ascending aorta alone (supra-commissural replacement) and the effects of prior operation were not taken into consideration. Each patient had a minimum of 2 follow-up CTA examinations following surgical repair with a time interval of 1–5 years (2.5 ± 1.3 years). The maximum diameter change between two follow-up CTA scans (minimal of 1 year) was measured and individual aortic growth rate was calculated. Patients were classified as unstable if the residual dissected aorta grew over 2.9 mm/year^24^. A total of 17 patients were analysed: stable (N = 9), and unstable (N = 8). Patient-specific CFD simulations were performed in order to obtain flow patterns, time-averaged wall shear stress (TAWSS) and pressure throughout the repaired TAAD.

### Anatomical features

**Figure 1:**
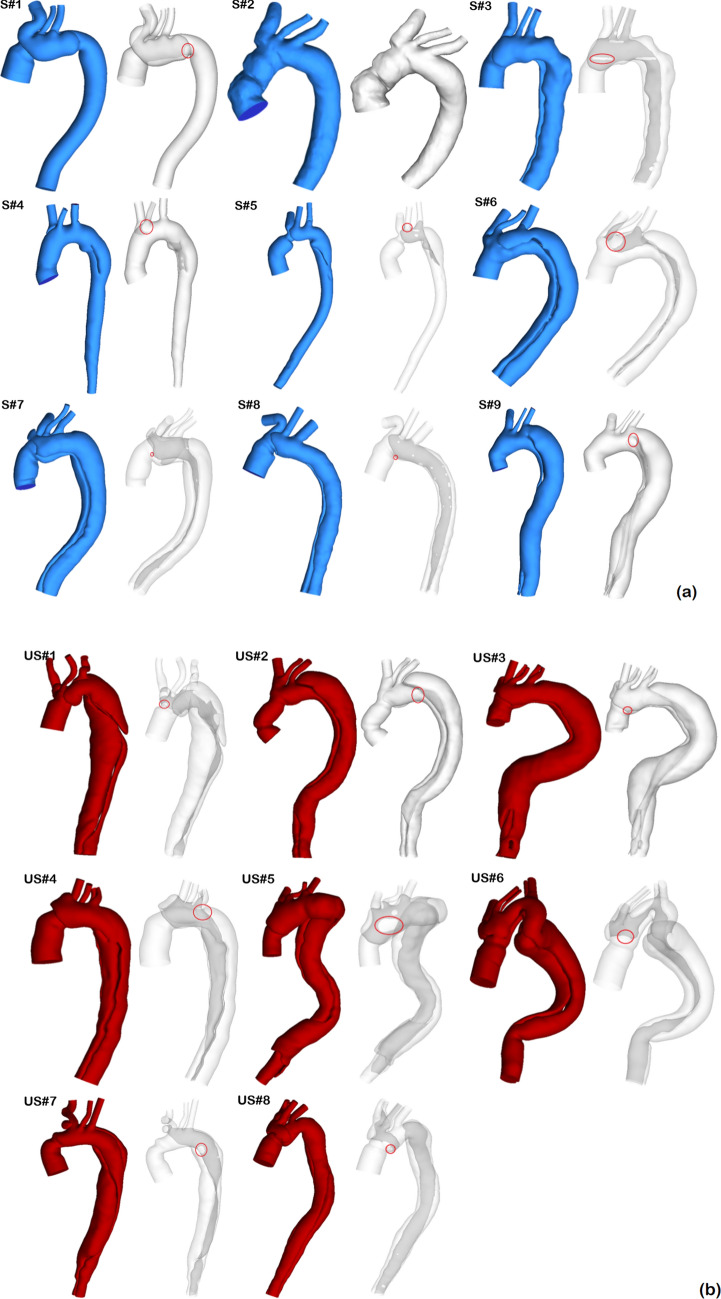
Geometric models of the 17 surgically repaired type-A aortic dissections reconstructed from computed tomography images. (**a**) Stable group, and (**b**) unstable group.

Figure 1 shows the geometric models reconstructed from the first set of post-surgery CTA images for all 17 patients. A transparent view for each model is also provided, which allows visualization of the location and shape of the tears. The location of the proximal entry tear is highlighted by a red circle in the transparent view. Among the 17 cases, only one patient (S1#2) had a fully thrombosed FL on follow up scan, while the others had a patent FL starting from the distal ascending aorta or the distal arch at the level of the left subclavian artery (LSCA). Three patients in the stable group had a short FL that merged with TL at the distal arch in one patient (S1#1), and in the mid-thoracic aorta of the other two (S1#4 and S1#5). A summary of key geometric parameters can be found in Table 1. In addition, statistical analyses of the evaluated parameters were performed using the non-parametric Mann–Whitney *U* test and a p-value < 0.05 was defined statistically significant. All results are presented as median [25 percentile, 75 percentiles] as summarised in Table 2.

**Table 1:** A summary of key geometric and hemodynamic parameters.

	Stable group (S)									Unstable group (US)							
	#1	#2	#3	#4	#5	#6	#7	#8	#9	#1	#2	#3	#4	#5	#6	#7	#8
**Geometric parameters**																	
Aortic growth rate ($\Delta D{\text{/year}}$) (mm/year)	1.2	1.2	1	1	0.3	0	0.5	0	2.2	8	4.8	5.7	3	3.4	3	3.1	14
Primary tear size																	
Area (mm^2^)	119	N/A	113	363	432	281	2.6	4.6	585	43	260	8.8	341	522	49	116	50
Number of re-entry tears	0	N/A	5	4	3	8	6	18	5	4	0	2	1	1	2	1	3
Distance between primary tear and LSCA* (mm)	− 30	N/A	38	34	33	37	32	35	− 6	70	− 33	39	− 8	34	56	− 29	29
Max. distance between tears (mm)	N/A	N/A	149	80	39	109	88	51	109	38	N/A	284	43	13	79	23	264
Max. aortic diameter (mm)	36	34	47	42	36	39	41	34	49	49	46	46	41	49	47	43	38
Dissection involved arch vessels (Y/N)	Y	N/A	Y	Y	Y	N	N	Y	Y	N	N	Y	Y	N	Y	N	Y
Inlet diameter (*d*_*1*_) (mm)	35	33	33	38	32	32	26	43	30	33	33	24	39	32	31	30	33
Arch diameter (*d*_*2*_) (mm)	24	28	38	36	31	35	35	22	43	30	43	34	36	35	26	30	36
Curvature ratio (*d*_*1*_*/d*_*2*_)	1.5	1.2	0.9	1.1	1.0	0.9	0.7	1.5	0.7	1.1	0.8	0.7	1.1	0.9	1.2	1.0	0.9
Tortuosity																	
Entire aorta (inlet to diaphragm)	2.0	2.7	2.1	2.4	2.4	2.3	2.4	1.6	2.2	2.1	2.9	2.4	2.0	2.7	3.0	1.9	2.2
Ascending aorta (inlet to LSCA)	1.1	1.3	1.2	1.1	1.2	1.1	1.2	1.2	1.2	1.1	1.2	1.3	1.3	1.3	1.2	1.3	1.1
Descending aorta (LSCA to diaphragm)	1.3	1.3	1.1	1.2	1.1	1.3	1.3	1.2	1.2	1.1	1.4	1.5	1.1	1.3	1.5	1.1	1.3
Volume ratio of FL^†^/TL^‡^	1.1	N/A	2	7.2	1.9	1.4	1.5	1.2	13.2	6.8	2.5	4.9	2.8	2.2	1.8	3.1	2.8
**Hemodynamic parameters**																	
Max. TAWSS^§^ (Pa)	7	12	7	6	13	13	25	25	13	18	16	33	22	5	32	23	45
Max. pressure difference (mmHg)	0.5	N/A	0.6	0.3	0.5	2.3	2.8	2.2	1.2	3.1	6.2	12	4	7.6	6.3	7	47

^†^False lumen; ^‡^True lumen; *Left subclavian artery; ^§^Time-averaged wall shear stress. Y and N refer to yes and no, respectively, while N/A means not known.

**Table 2:** Comparison of geometric and hemodynamic parameters between the two groups.

	Stable group (S)	Unstable group (US)	Mann–Whitney *U* test
	Median [Q1, Q3]	Median [Q1, Q3]	P-value
**Geometric parameters**			
Aortic growth (mm/year)	1 [0.2, 1.2]	4.1 [3, 7.4]	0.001
Primary tear size			
Area (mm^2^)	200 [31.7, 414.8]	83 [44.5, 320.8]	0.6
Number of re-entry tears	5 [3.3, 7.5]	1.5 [1, 2.8]	0.02
Distance between primary tear to LSCA* (mm)	33.5 [3.5, 36.5]	31.5 [− 23.8, 51.8]	0.8
Max. distance between tears (mm)	88 [51, 109]	43 [23, 264]	0.3
Maximum aortic diameter (mm)	39 [35, 44]	46 [41.5, 48.5]	0.07
Inlet diameter (*d*_*1*_) (mm)	33 [31, 36.5]	32.5 [30.3, 33]	0.6
Arch diameter (*d*_*2*_) (mm)	35 [26, 27]	34.5 [30, 36]	0.8
Curvature ratio (*d*_*1/*_*d*_*2*_)	1 [0.8, 1.4]	0.95 [0.83, 1.1]	0.6
Tortuosity			
Entire aorta (inlet to diaphragm)	2.3 [2.1, 2.4]	2.3 [2, 2.9]	0.6
Ascending aorta (inlet to LSCA)	1.2 [1.1, 1.2]	1.3 [1.1, 1.3]	0.2
Descending aorta (LSCA to diaphragm)	1.2 [1.2, 1.3]	1.3 [1.1, 1.5]	0.4
Volume ratio of FL^†^/TL^‡^	1.7 [1.3, 5.9]	2.8 [2.3, 4.5]	0.14
**Hemodynamic parameters**			
Max. TAWSS^§^ (Pa)	13 [7, 19]	22.5 [16.5, 32.8]	0.07
Max. pressure difference (mmHg)	0.9 [0.5, 2.3]	6.7 [4.6, 10.9]	0.001

^†^ False lumen; ^‡^True lumen; *Left subclavian artery; ^§^Time-averaged wall shear stress.

Distance between the primary entry tear and the origin of LSCA was estimated by measuring the respective aortic centreline. The primary tear was observed in the proximal arch in 11 patients, whereas in 5 cases the primary tear was located distally to the LSCA as indicated by the negative values. Residual primary entry tears varied in size and shape from patient to patient, the smallest and largest tears were 2.6 mm^2^ and 585 mm^2^, respectively, both in the stable group. In the 16 patients with a patent FL, the TL was compressed by the FL, with the FL/TL volume ratio ranging from 1.1–13.2 in the stable group, and 1.8–6.8 in unstable group. The overall FL/TL volume ratio was higher in the unstable group with 7/8 (88%) patients had a value greater than 2, while only 2/8 (25%) patients in the stable group had a value greater than 2. Nevertheless, the two groups neither differ significantly in primary tear size (Stable group, 200 [31.7, 414.8] vs. Unstable group, 83 [44.5, 320.8] mm^2^; P = 0.60) nor in FL/TL volume ratio (1.7 [1.3, 5.9] vs. 2.8 [2.3, 4.5]; P = 0.14).

Obviously, the stable group had a much lower aortic growth rate than the unstable group (1 [0.2, 1.2] vs. 4.1 [3, 7.4] mm/year; P = 0.001). The number of re-entry tears varied from 0 to 18 in the stable group, compared to 0 to 4 in the unstable group. In the patient with no re-entry fenestration distal to the proximal tear (S1#1), the residual false lumen involved the aortic arch and extended to the innominate artery. Patients in the stable group were found to have a significantly larger number of re-entry tears than the unstable group (5 [3.3, 7.5] vs. 1.5 [1, 2.8]; P = 0.02). In addition, patients in the stable group had smaller maximum aortic diameters than the unstable group (39 [35, 44] vs. 46 [41.5, 48.5] mm; P = 0.07), but the difference was not statistically significant. No obvious differences were found for other geometric parameters between the two groups (Table 2).

### Flow patterns

**Figure 2:**
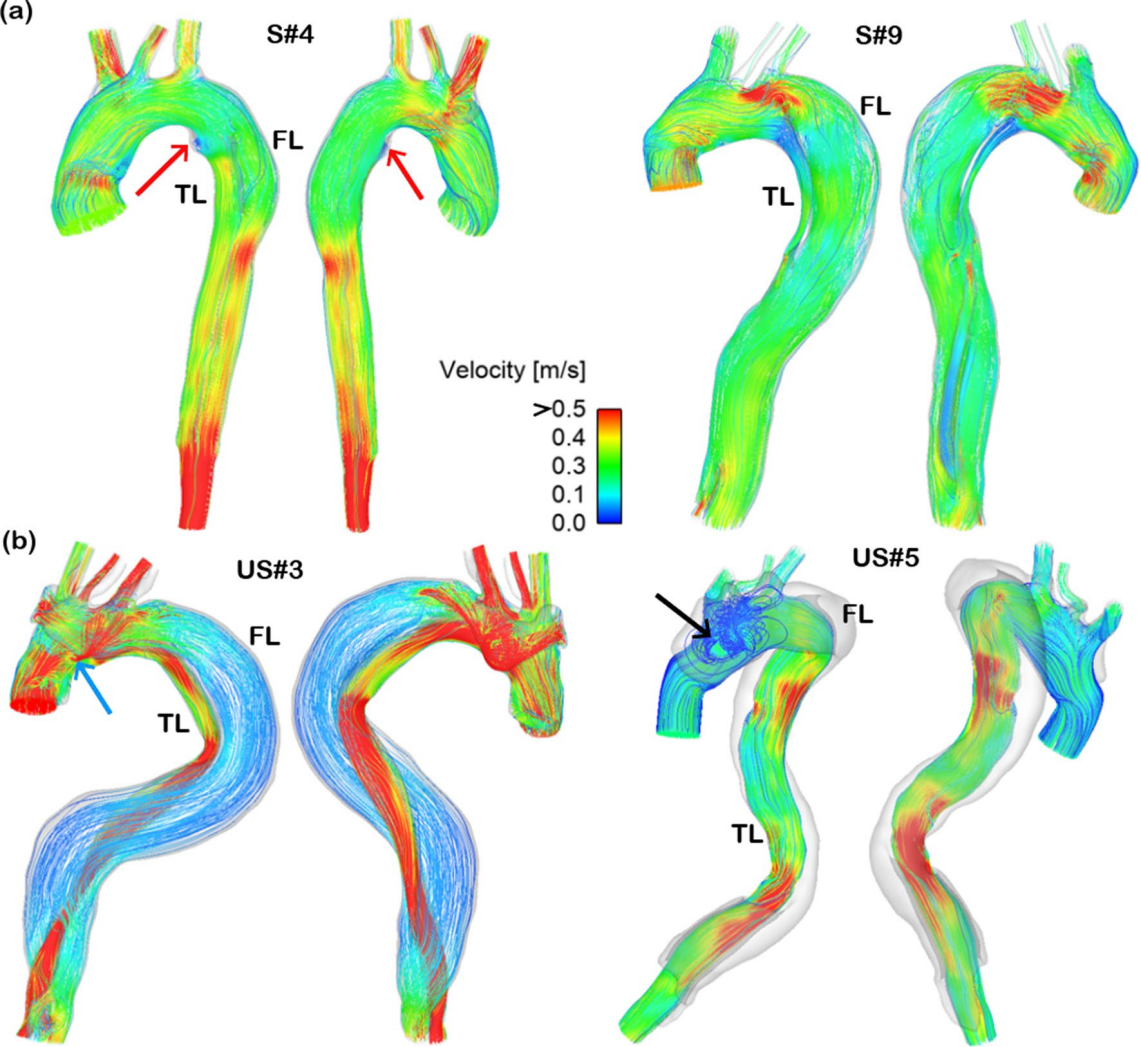
Instantaneous velocity streamlines for four selected cases at peak systole. (**a**) Two cases from the stable group, and (**b**) two cases from the unstable group. Velocities > 0.5 m/s are shown in red. In patient S#4, the merged true lumen (TL) and false lumen (FL) in the mid-descending aorta resulted in a reduced downstream luminal area causing flow acceleration. The presence of a sudden expansion in the proximal TL led to recirculating flow with low velocities (red arrow). In patient S#9, despite TL compression (FL/TL ratio: 13.2), flow acceleration did not occur in the narrowed lumen area as the blood flow was drawn anteriorly by the FL due to large entry tear. In contrast, blood was greatly accelerated by the compressed TL in patient US#3. Patient US#3 presented with a small entry tear inducing a high-velocity jet (blue arrow). However, this jet was dissipated downstream due to the expanding FL and absence of distal communications. In patient US#5, very small flow entered into FL with stagnation and recirculation (black arrow).

Instantaneous velocity streamlines at peak systole in 4 cases (2 from each group) are displayed in Fig. 2. In general, the TL and FL had comparable blood velocities for patients in the stable group, while much higher blood velocities were observed in the TL of the unstable group. Although there were substantial individual variations, some common flow characteristics could still be observed. Owing to geometric complexity, flow patterns were dominated by strong helical flow, flow separation and recirculation. Accelerated flow could be seen in regions with reduced lumen areas, especially at the primary tear which caused the formation of a high-velocity jet. Flow patterns for all the other patients are shown in supplementary information (S1).

### Time-averaged wall shear stress (TAWSS)

**Figure 3:**
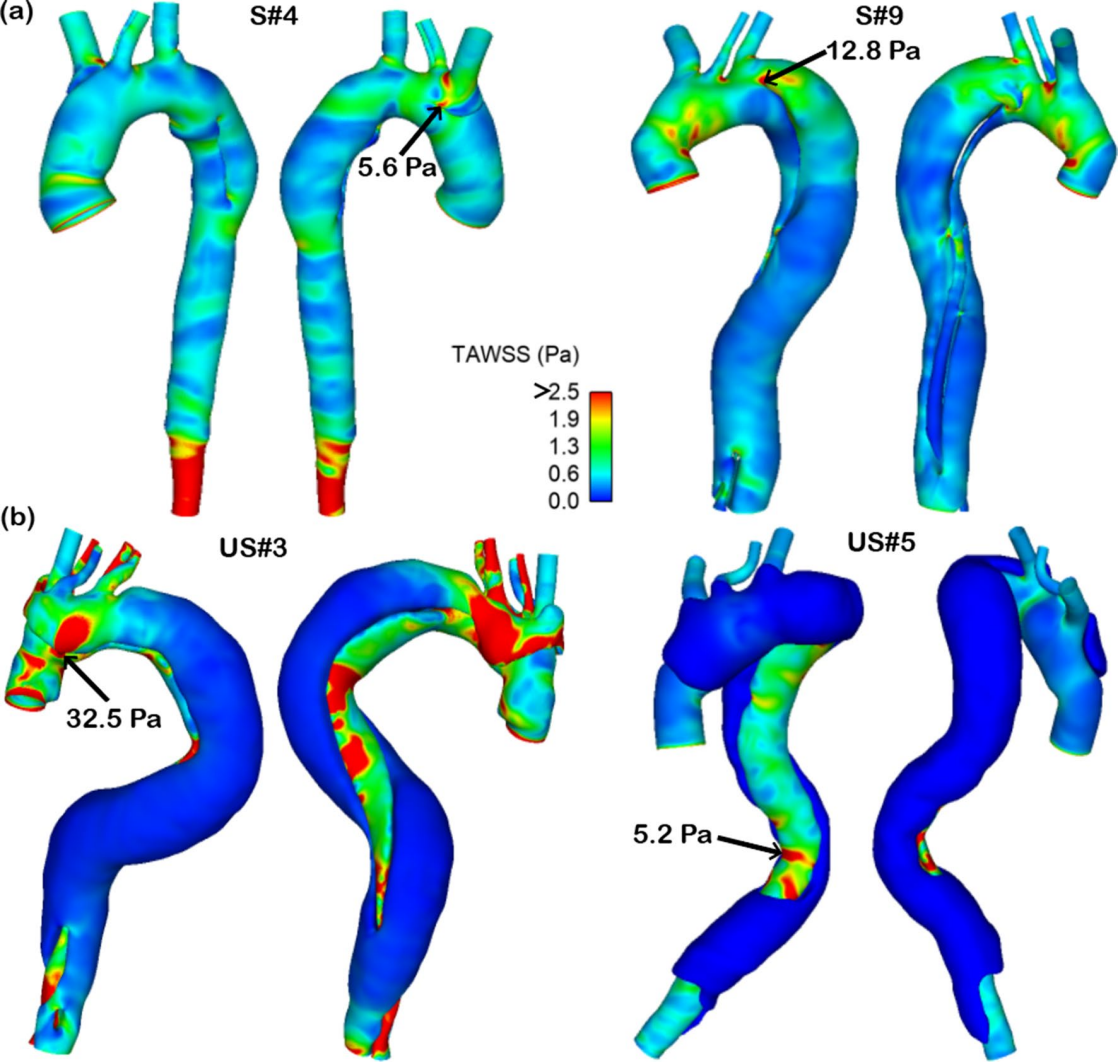
Comparison of time-averaged wall shear stress (TAWSS) in selected patients. (**a**) Stable group, and (**b**) unstable group. The images show that the TAWSS is comparable in the true lumen (TL) and the false lumen (FL) in the stable group, but significantly different in the unstable group. TAWSS > 2.5 is shown in red and low TAWSS is shown in blue. In most cases, the maximum TAWSS was in regions around the entry tears (black arrows), except for patient US#5, whose entry tear was in a transverse position so that very low flow passed into the FL.

Wall shear stress is the frictional force exerted by blood flow on the luminal surface, which can be calculated based on velocity gradient at the wall and blood viscosity. Using the resolved flow field, instantaneous WSS values were calculated and their time-averaged values were obtained. Figure 3 shows the TAWSS contours in the same representative examples as in Fig. 2, where regions with TAWSS > 2.5 Pa are shown in red colour. Overall, the magnitude of TAWSS was comparable between the TL and FL for the stable group, but in the unstable group TAWSS was higher in the TL. Much higher TAWSS were observed in regions surrounding the tears, with the maximum TAWSS on the edge of the tears in most cases (Fig. 3). Comparison of the maximum TAWSS between the two groups revealed higher maximum TAWSS in the unstable group than the stable group (13 [7, 19] vs. 22.5 [16.5, 32.8] Pa; P = 0.07) (Table 2). TAWSS distributions for all the patients can be found in Supplementary Material (S2).

### Pressure difference between true and false lumen

**Figure 4:**
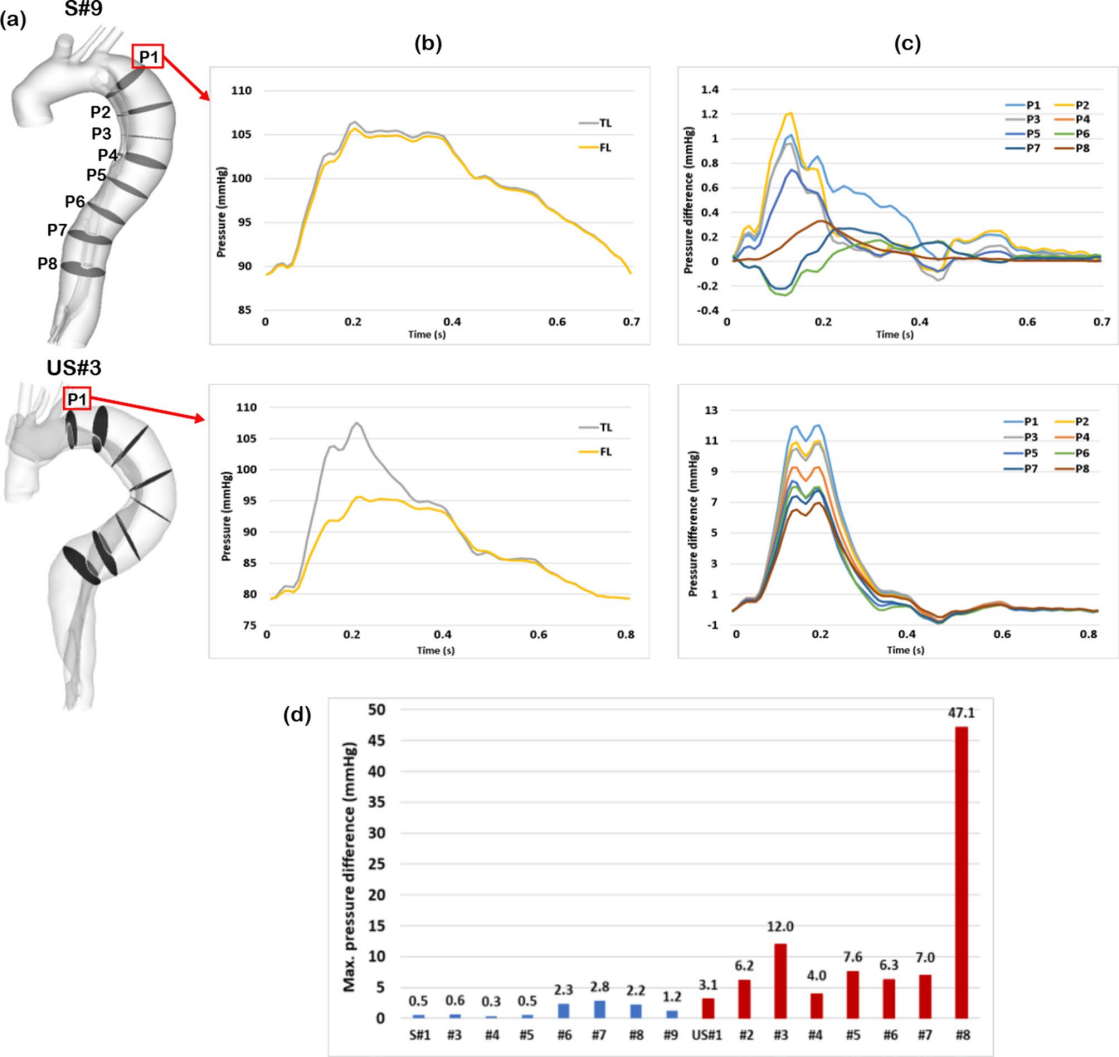
Evaluation of pressure difference between the true and false lumen. (**a**) Eight cross-sectional planes were created along the centrelines of the dissected aorta, (**b**) pressure waveforms at a selected cross-section plane, such as P1, were calculated for both the true and false lumen, (**c**) pressure difference (*P*_*TL*_* – P*_*FL*_) was further determined based on the evaluated true and false lumen pressure waveforms, and (**d**) absolute values ($\left| {P_{TL} - P_{FL} } \right|$) of the maximum pressure differences over a cardiac cycle at all cross- sectional planes were then evaluated for each patient.

In order to calculate pressure difference between the true and false lumen, eight cross-sectional planes were selected along the centrelines of the dissected aorta, with P1 being 2 cm from the origin of LSCA and P2–P8 being evenly spaced below P1 with an interval of 3 cm (Fig. 4a). Pressure variations for both the true and false lumen over a cardiac cycle at cross-sectional plane 1 are shown in Fig. 4b for two representative cases, one from each group. It can be seen that pressures in the TL and FL were almost identical for S#9, but notable difference can be seen in US#3. The evaluated TL and FL pressures were further analysed by examining the pressure difference (*P*_*TL *_*–* *P*_*FL*_) over a cardiac cycle (Fig. 4c). It should be mentioned that fewer cross-sectional planes could be created in 3 patients in the stable group due to their short FL. The maximum TL and FL pressure differences among all cross-sectional planes were then evaluated for each patient (Fig. 4d) and the absolute values ($\left| {P_{TL} - P_{FL} } \right|$) are reported in Table 1. Based on statistical analysis (Table 2), luminal pressure difference was significantly different between the two groups (0.9 [0.5, 2.3] vs. 6.7 [4.6, 10.9] mmHg; P = 0.001).

## Discussion

TAAD is a life-threatening cardiovascular disease that usually requires urgent open surgery since the mortality rate of untreated patients is about 50% within the first 48 h^25^. The standard surgical approach for TAAD is supra-commissural replacement of the ascending aorta with synthetic tube grafts^26^. Following a supra-commissural ascending aortic repair in TAAD, the residual dissected segments may continue to dilate and rupture^1^. With a persistent patent FL, 20–50% of patients are reported to die within 5 years, and 40–70% within 10 years^27–29^. This study evaluates the morphological and hemodynamic features that may predict progressive aortic dissection based on patient-specific anatomical data and CFD simulation results. We used standard CTA images for the CFD assessment which makes the technique applicable to routine clinical practice.

The study cohort contained 17 patients (9 stable and 8 unstable), among these, one patient had a fully thrombosed FL after surgery, while all the other patients maintained a patent FL. A fully thrombosed FL after surgery is expected to have an improved outcome^30^. The aortic growth rate (diameter change per year) was significantly different between the patients with a stable aorta (1 [0.2, 1.2] mm/year) and those with unstable aortic growth (4.1 [3, 7.4] mm/year). Reintervention is usually recommended for patients with a growth rate of greater than 3 mm per year^24^. Patients with stable aortic diameters were found to have statistically significant larger number of re-entry tears (5 [3.3, 7.5]) than those with progressive aortic dilatation (1.5 [1, 2.8]). The lack of distal tears has been reported to increase FL pressure^19–21,31^. Wan et al. suggested that additional entry tears would reduce the TL/FL pressure difference and prevent FL expansion and TL collapse^16^. A clinical study also showed that communications between the TL and FL through multiple distal re-entry tears might prevent TL collapse and should be a positive prognostic sign^30^.

Previous studies on TBAD found that larger entry tears would result in greater flow entering the FL^10,22^, which could lead to rapid aneurysmal expansion^19^. However, neither entry tear size nor FL flow were found to be significantly different between the two groups in our study. Aortic geometries included in the present study are more complex than those reported in previous TBAD models, as most of the entry tears were located in the ascending aorta or arch rather than distal to the LSCA. Therefore, the amount of flow entering the FL would depend not only on the entry tear size but also its orientation relative to the flow direction. Moreover, it has been reported that patients presented with multiple distal tears have increased FL flow^32^. This may explain why some patients with very small entry tears but multiple communication tears were found to have relatively high flow in the FL.

Other evaluated anatomical features included FL/TL volume ratio and the tortuosity of the aorta, but none of these were able to statistically differentiate the two groups. Another morphological parameter of interest was the maximum aortic diameter. Although the maximum aortic diameter was larger in the unstable group (46 [41.5, 48.5] mm) compared to the stable group (39 [35, 44] mm), the evidence was weak in this small cohort. Nowadays, clinicians mainly rely on the maximum aortic diameter to predict aortic dilatation and its risk of rupture. The reported median rupture size for the ascending thoracic aortic aneurysms (TAAs) is approximately 6 cm, which is much lower than 7.2 cm for descending TAAs^33^. However, previous studies showed that maximum diameter alone was not a reliable predictor of late complications^34–36^. Our study shows that in addition to sizing criteria, haemodynamic parameters should be considered when addressing the risk of aortic dilatation.

Blood flow was highly disturbed in all repaired TAAD models with varying extent of flow recirculation in both true and false lumens, especially in the region surrounding the tear. Large recirculation regions have been associated with thrombus formation, while partial thrombosis in the FL might occlude distal tears, impede outflow and therefore increase the risk of death from further aortic dilatation and rupture^30^. Abnormal high WSS is associated with endothelial damage^37^ and degenerative lesions of the dissected wall, leading to wall weakening and eventual rupture^38^. Regions of high TAWSS were observed in the opposing FL wall as a result of direct impingement of jet flow when passing through the tear. Elevated TAWSS in the FL has been suggested to cause subsequent increase in luminal diameter^34^. In most of the cases, the maximum TAWSS was found around the primary tear, perhaps indicating the vulnerability of this region to further increase in tear size. Our results showed that patients in the unstable group had higher maximum TAWSS on the aortic wall, similar to those reported for type B dissection^19^, although this finding was not statistically significant. It was noted that two outliers in the stable group (S#7 and S#8) had relatively high maximum TAWSS due to the existence of very small entry tears. Small tears have been reported to result in higher tear velocities^39^ and therefore increased wall shear stress. In the unstable group, very low peak TAWSS was found for patient US#5, whose entry tear had a transverse orientation allowing a very small amount of flow entering the FL with low velocities.

Pressure difference between the true and false lumen is a key factor in driving the deformation of both lumens. Previous small case series reported that higher pressure difference is associated with further aneurysmal dilatation in type B dissections^20,22^, but no similar studies on TAAD could be found in the literature. We demonstrated that patients with progressive aortic dilatation following TAAD repair had significantly higher luminal pressure difference (6.7 [4.6, 10.9] mmHg) compared to the stable group (0.9 [0.5, 2.3] mmHg). It is also of interest to note that pressure difference between the true and false lumen is influenced by the number and size of tears^31,32^. As mentioned before, multiple tears along the length of the dissection lead to an equalisation of pressure between two lumens. In an in vitro study of TBAD, a smaller tear was found to cause a greater luminal pressure difference, whereas larger tears led to equalised true and false lumen pressures^39^. This was not exactly true based on the results presented here, for example, 2 patients in the stable group (S#7 and S#8) with very small entry tears did not present with large pressure imbalance between the true and false lumen. The effect of small entry tears on luminal pressure difference was counteracted by the presence of multiple re-entry tears. In contrast, a patient (US#5) with a large primary entry tear had a considerably high pressure difference as flow entering the FL appeared to be unidirectional with no exit to relieve the pressure. As reported in the literature^40^, in the case of blind end of the FL, absence of a distal tear or a side branch to shunt pressure from the FL may result in pressure difference between TL and FL.

The main limitation of this study is the small sample size that may limit wider applicability of the findings. Individual aortic growth rate was calculated by dividing the change in aortic diameter between the initial and follow up scans by the corresponding time interval. This might not be accurate and a more complex mixed effect model with repeated measurement of aortic diameters should be considered in the future. With regard to the computational model, the rigid wall assumption may miss the opportunity to capture some complex flow structures caused by intimal flap motion^41^. This assumption can be relaxed by performing a fluid–structure interaction (FSI) simulation, which couples the flow model with a compliant wall model. However, building a compliant wall model of aortic dissection requires information on wall thickness and material properties which are difficult to measure in vivo. Additionally, FSI simulations are computationally expensive, which will limit its use in comparative studies involving a large cohort of patients. Finally, patient-specific pressure data were not available due to retrospective nature of the study, which may compromise the patient specificity of the predicted pressure values but should not affect the evaluated pressure difference^42^. It will be of interest to perform serial CFD assessment of the available imaging data during the follow-up period to assess changes and their impact on clinical outcome.

## Conclusion

The number of re-entry tears, as well as pressure difference between the true and false lumen were found to be significantly different between the patients with stable and unstable aortic diameters following TAAD surgical repair. Patients with unstable aortic dilatation were found to have higher luminal pressure differences but fewer number of re-entry tears. Although not statistically significant based on our data, large maximum aortic diameter as well as high maximum TAWSS on the aortic wall might also contribute to rapid aneurysmal expansion. These findings are promising and point to a potential role of hemodynamic factors in determining the risk of progressive aortic dilatation following TAAD repair. Further large cohort studies are warranted to validate these findings.

## Methods

### Study design

All procedures performed in studies involving human participants were in accordance with the ethical standards of the institutional and/or national research committee and with the 1964 Helsinki declaration and its later amendments or comparable ethical standards. Ethic approval was obtained on 04/05/2020 from the Institutional committee of Health Research Authority (HRA) and Health and Care Research Wales (HCRW), with a REC reference of 20/WM/0145, and need for informed consent was waived.

Patient-specific dissection geometries were reconstructed from post-dissection repair computed tomography angiography (CTA) images and divided into two groups, patients with stable aortic diameters, and those with progressive aortic dilatation. These were then coupled with physiologically realistic boundary conditions. Hemodynamic results including flow patterns, time-averaged wall shear stress (TAWSS) and pressures were compared.

### Data acquisition

A total of 17 patients were included in this study. All patients underwent a minimum of 2 follow-up CTA examinations following surgical repair with a time interval of 1–5 years (2.5 ± 1.3 years). All studies were reviewed by a radiologist with over 10-year experience in cardiovascular imaging. The changes in total aortic diameter were assessed using double oblique multiplanar reconstruction at the following locations: (a) the mid and distal aortic arch, and (b) the proximal, mid and distal descending thoracic aorta. The maximum diameter change among different locations was then used to calculate the individual aortic growth rate by dividing the maximum difference in diameter by the time interval between two follow-up CTA scans (minimal of 1 year). Patients were classified as unstable if the residual dissected aorta grew over 2.9 mm/year^24^.

### Geometry reconstruction and morphological measurements

**Figure 5:**
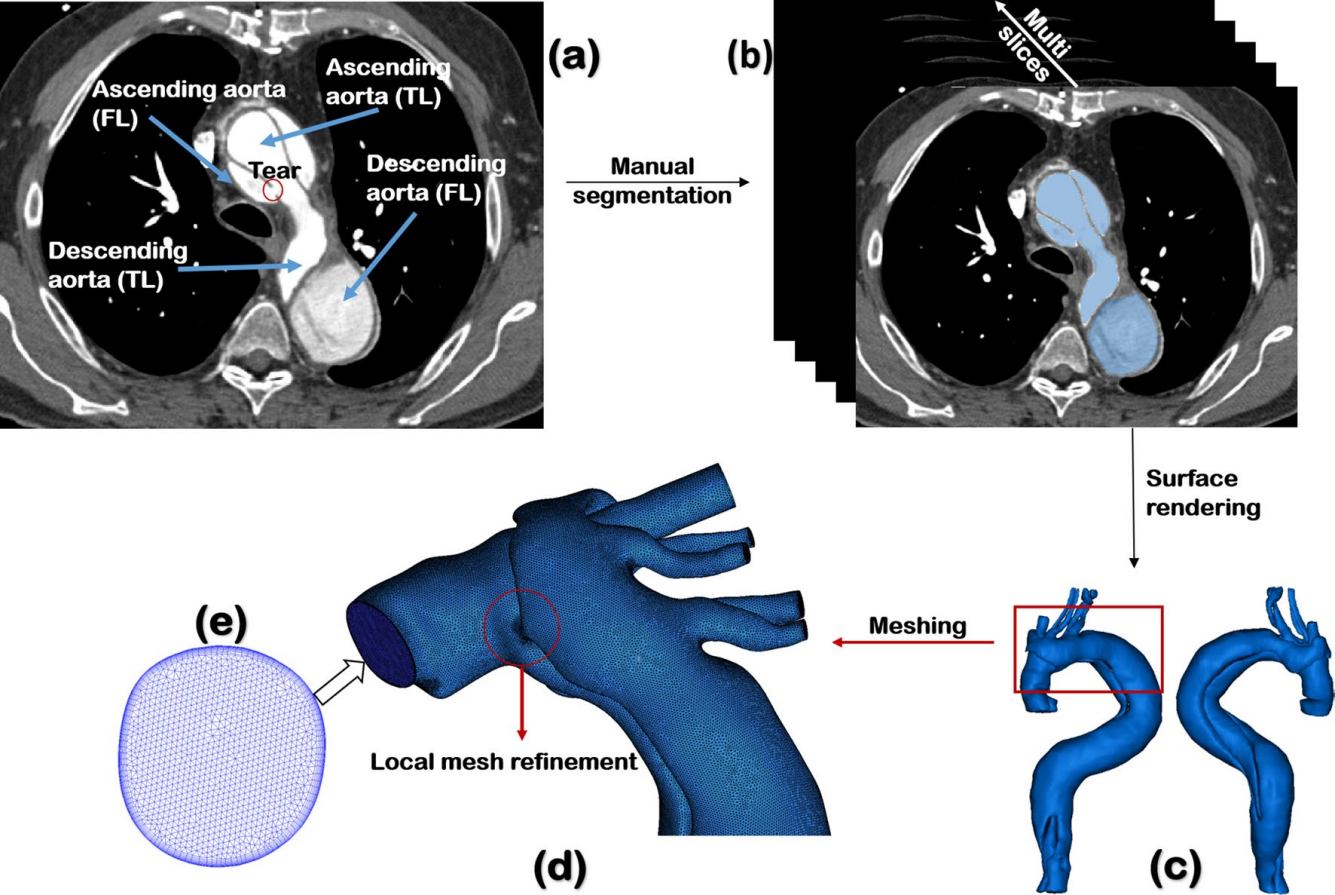
Workflow of patient-specific geometry reconstruction from computed tomography (CT) scans to mesh generation. (**a**) Target lumen areas are identified on CT, (**b**) cross-sectional slices were manually segmented to separate the true and false lumen, (**c**) dissection geometry is defined as the fluid domain of interest. All aortic geometries were smoothed and cut out from the sinotubular junction to the level of diaphragm, including the arch vessels. (**d**) Meshing, where the fluid domain was discretised into a large number of numerical grids with local mesh refinement performed, and (**e**) hybrid unstructured mesh comprising of a tetrahedral core and prismatic wall layers.

CTA data were obtained with a multi-slice computed tomography scanner (Somatom Definition Flash, Siemens Medical Solutions, Germany). The quality of CT images was sufficient to include all the important geometric features, with slice thickness and increment ranging from 0.63–1 mm and 0.5–0.8 mm, respectively. All patient-specific geometries were reconstructed from the first set of CTA data using a semi-automatic threshold-based segmentation tool (Mimics 20.0, Materialise HQ, Leuven, Belgium). As shown in Fig. 5a, several essential anatomical features can be recognised clearly from the CT images, including the dissection tears, intimal flap, and the true and false lumen. The regions of interest had to be manually segmented based on the local greyscale intensity (Fig. 5b). The segmented 2D masks were then integrated to generate a 3D fluid domain, which was smoothed to eliminate any reconstruction errors. 3-D surface smoothing was performed by using a cubic spline algorithm. For every patient, the computed region was created from the aortic sinotubular to the level of diaphragm. Three main arch branches were also included in the reconstructions (Fig. 5c). Since post-surgical dilatations mainly occurred in the thoracic aorta, the abdominal aorta was excluded from the analysis to reduce the computational time.

All the 3D geometries were then imported into ANSYS ICEM CFD (ANSYS, Canonsburg, PA, US) to generate computational mesh, which involved local refinement in regions around the tears and great curvatures (Fig. 5d). The mesh consisted of tetrahedral elements in the core and 10 layers of prismatic cells near the wall (Fig. 5e). Mesh sensitivity tests were conducted, and the number of elements adopted in the final analysis ranged from 3 to 7.4 million, depending on geometric complexity and size of the model.

After geometry reconstruction, a number of morphological measurements were taken using Mimics software including distances, best-fit diameters, and volumes, as summarised in Table 1. It should be mentioned that tortuosity was defined as a ratio between the distance along the centreline and the linear distance, which was calculated for the entire aorta, and for the ascending and descending aortas separately. The re-entry tears were identified on the CTA images as openings in the dissection membrane that were located distal to the primary entry tear. It should be noted that the ‘primary entry tear’ referred to in this paper is the most proximal tear in the residual dissected aorta since the initial primary tear in the ascending aorta has been resected during surgery.

### Boundary conditions

**Figure 6:**
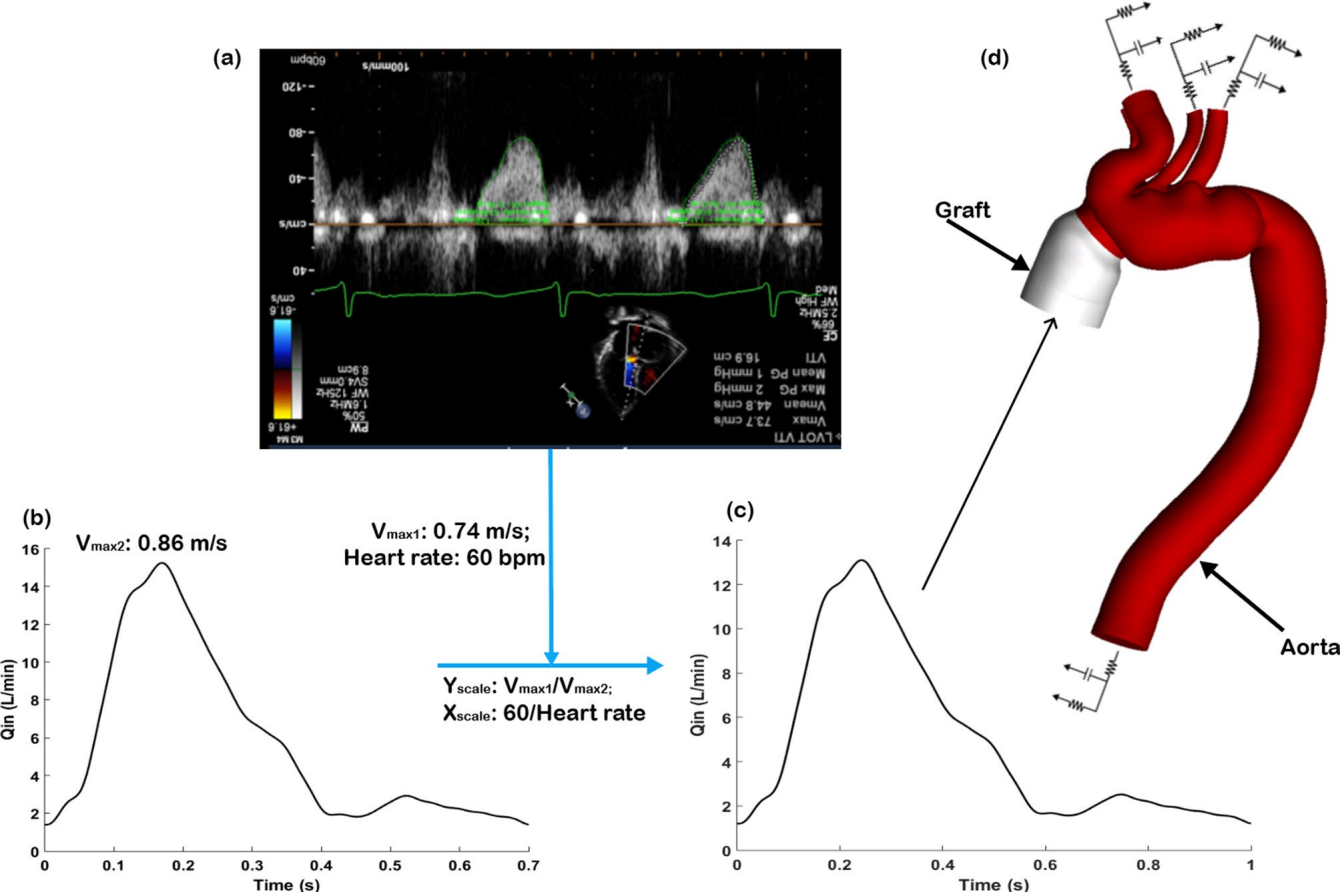
Physiological boundary conditions for patient-specific flow simulations. (**a**) Velocity measurements acquired by Doppler echocardiography could not be applied directly due to the absence of diastolic velocity data, (**b**) in vivo measured flow waveform acquired at a similar location of the ascending aorta of a type B dissection was taken from the literature^43^, (**c**) scaled flow waveform based on patient-specific heart rate and maximum inlet velocity, and (**d**) schematic of the computational model employed in this study. The scaled patient-specific flow waveform was prescribed at the model inlet while a 3-element Windkessel model was applied for all the outlets. Graft (light grey) and the aorta (red) are indicated by black arrows.

As shown in Fig. 6, physiological boundary conditions were applied in order to generate results that are clinically relevant. In this study, although Doppler ultrasound measurements of patient-specific velocity waveforms (Fig. 6a) were available, this information could not be directly employed due to the absence of diastolic velocity data. Furthermore, velocity data were acquired at the left ventricular outflow tract (LVOT) rather than at the model inlet. Considering the above limitations, in vivo measured flow waveform acquired at a similar location in the ascending aorta of a type B aortic dissection patient was adopted as a template^43^, and the corresponding maximum velocity was recorded as shown in Fig. 6b. This flow waveform was then scaled in both directions by making use of patient-specific heart rate and maximum velocity measured by Doppler ultrasound, so that the applied flow waveform (Fig. 6c) contained some patient-specific features. The individually scaled flow waveform was specified at each model inlet along with the assumption of a flat velocity profile. With regard to outlet boundary conditions, a total of 21% of inlet flow was assumed to exit through the arch vessels^43^, where flow split was calculated based on their cross-sectional areas^44^. This information was used to calculate parameters in a 3-EWM which was implemented at each model outlet (Fig. 6d). The assumption of rigid vessel walls was made where no-slip boundary conditions were specified. This assumption was not unreasonable given the reduced compliance of dissected aortic walls.

### CFD model

Following mesh generation, blood flow through these patient-specific models were simulated by solving the Navier–Stokes equations (Eqs. 1 and 2) with a finite volume based solver (ANSYS CFX 15, ANSYS, Canonsburg, PA, US).

1 $$ \nabla \cdot (\rho {\mathbf{u}}) = 0 $$

2 $$ \frac{{\partial (\rho {\mathbf{u}})}}{\partial t} + \nabla \cdot (\rho {\mathbf{uu}}) = - \nabla p + \mu \nabla^{2} {\mathbf{u}} $$

where $\rho$ is the blood density, $\nabla$ is the divergence operator, ${\mathbf{u}}$ is the velocity vector, ${\text{p}}$ is the pressure, and $\mu$ is the viscosity of blood. Here, blood was assumed to be incompressible and Newtonian with a constant density of 1060 kg/m^3^ and dynamic viscosity of 4 mPa·s.

Cycle-averaged Reynolds numbers were in the range of 589 and 1585, while the peak values were between 1620 and 4035, which were calculated based on the inlet diameter and the mean and peak velocities of the reconstructed aorta models, respectively. Flow in a dissected aorta is likely to become transitional or turbulent induced by geometric features, such as a narrow tear or highly compressed TL. In order to capture any possible flow turbulence, a shear stress transport (SST-Tran) model^45^ was applied. A fixed time step of 0.001 s was chosen, and a maximum root-mean-square residual of 1 × 10^–5^ was specified as a convergence criterion. The period of one cardiac cycle ranged from 0.46 to 1 s based on the patients’ heart rate. All simulations were performed for a minimum of three cardiac cycles to achieve a periodic solution, and results obtained in the last cycle were used for detailed analysis. Flow patterns, TAWSS as well as pressure were calculated and analysed using CEI Ensight 10 (CEI Inc, Apex, NC, US).

### Statistics

Statistical analysis was carried out using SSPS v. 23.0 (IBM Corp., Armonk, NY, USA). Due to the limited sample size, the non-parametric Mann–Whitney *U* test was used to determine if there were significant differences in the measured anatomical and haemodynamic parameters between the two groups. Distributions of each parameter were similar for the two groups, as assessed by visual inspection using SPSS. Therefore, all results were presented as median [25 percentile, 75 percentiles]. A p-value < 0.05 was considered statistically significant.

## Supplementary Information

Supplementary Figures.

## Data Availability

The datasets generated during and/or analysed during the current study are available from the corresponding author on reasonable request.
