# Supplementary material for: Association of hemodynamic factors and progressive aortic dilatation following type A aortic dissection surgical repair
Source: Sci Rep. 2021 Jun 1;11:11521. doi: 10.1038/s41598-021-91079-5 (PMC8169847; doi:10.1038/s41598-021-91079-5)
Supplement: Supplementary file 1 — Supplementary Figures. [file 41598_2021_91079_MOESM1_ESM.pdf]

Supplementary Information

Association of hemodynamic factors and progressive aortic dilatation following type A aortic dissection surgical repair

Yu Zhu[1], Saeed Mirsadraee[2], George Asimakopoulos[3], Alessia Gambaro[4], Ulrich Rosendahl[3], John Pepper[3], Xiao Yun Xu[1,*]

[1]Department of Chemical Engineering, Imperial College London, London SW7 2AZ, United Kingdom

[2]Department of Radiology, Royal Brompton and Harefield Hospitals NHS Trust, London SW3 6NP, United Kingdom

[3]Department of Cardiac Surgery, Royal Brompton and Harefield Hospitals NHS Trust, London SW3 6NP, United Kingdom

[4]Department of Cardiology, Royal Brompton and Harefield Hospitals NHS Trust, London SW3 6NP, United Kingdom

Correspondence and requests for materials should be addressed to X.Y.X (yun.xu@imperial.ac.uk).

S1. Flow patterns

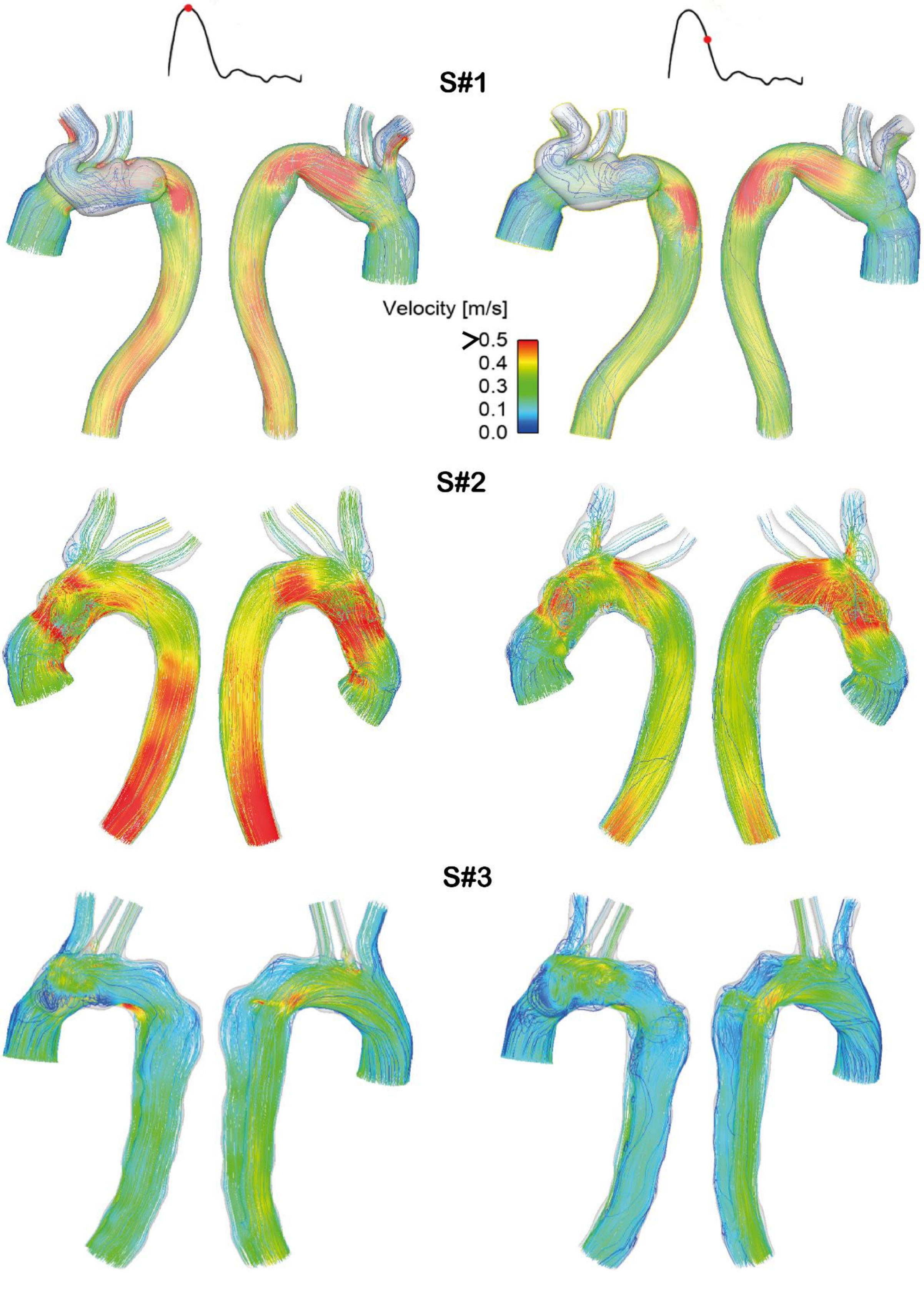

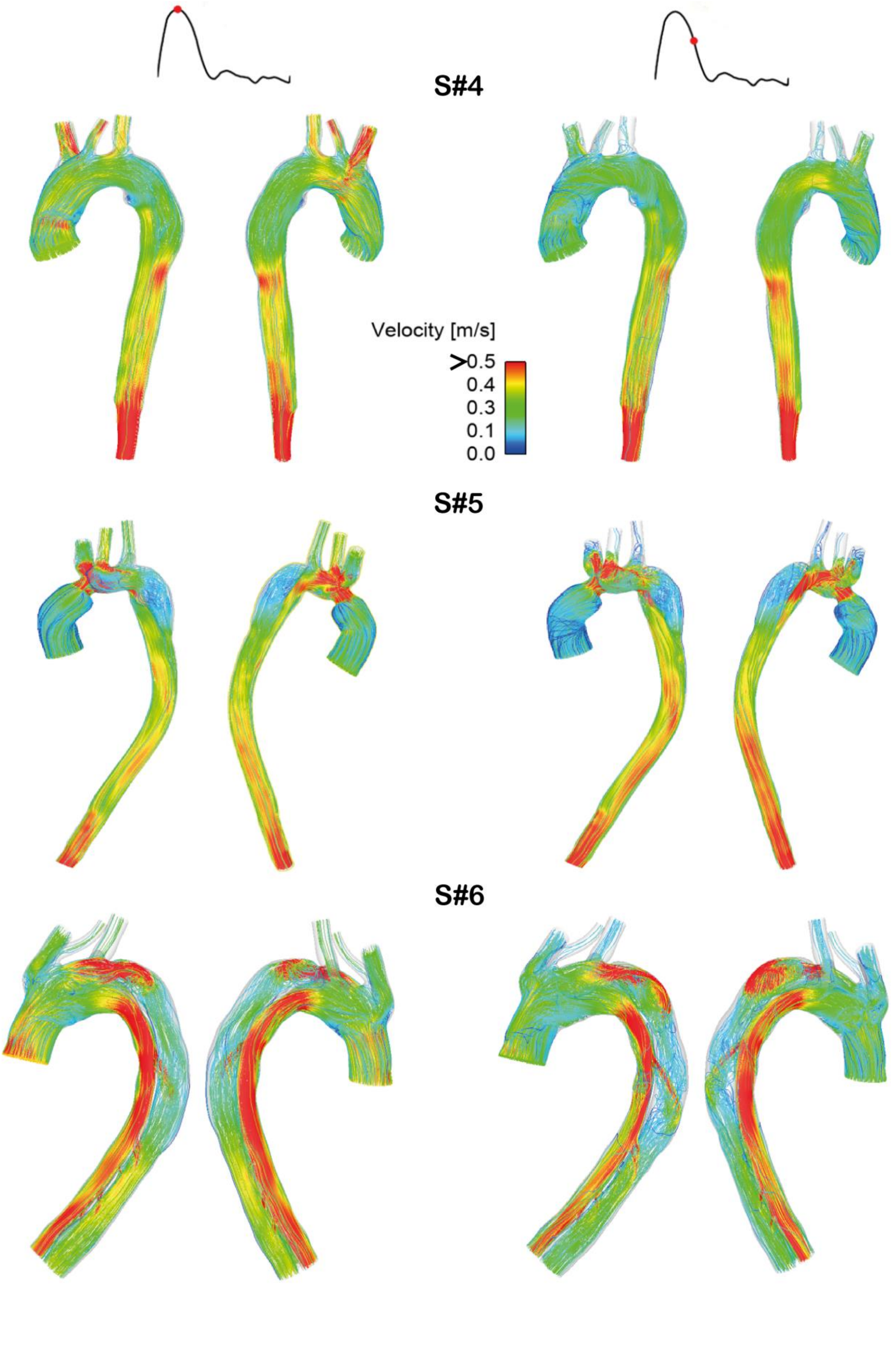
S#4
Velocity [m/s]
>0.5
0.4
0.3
0.1
0.0
S#5
S#6

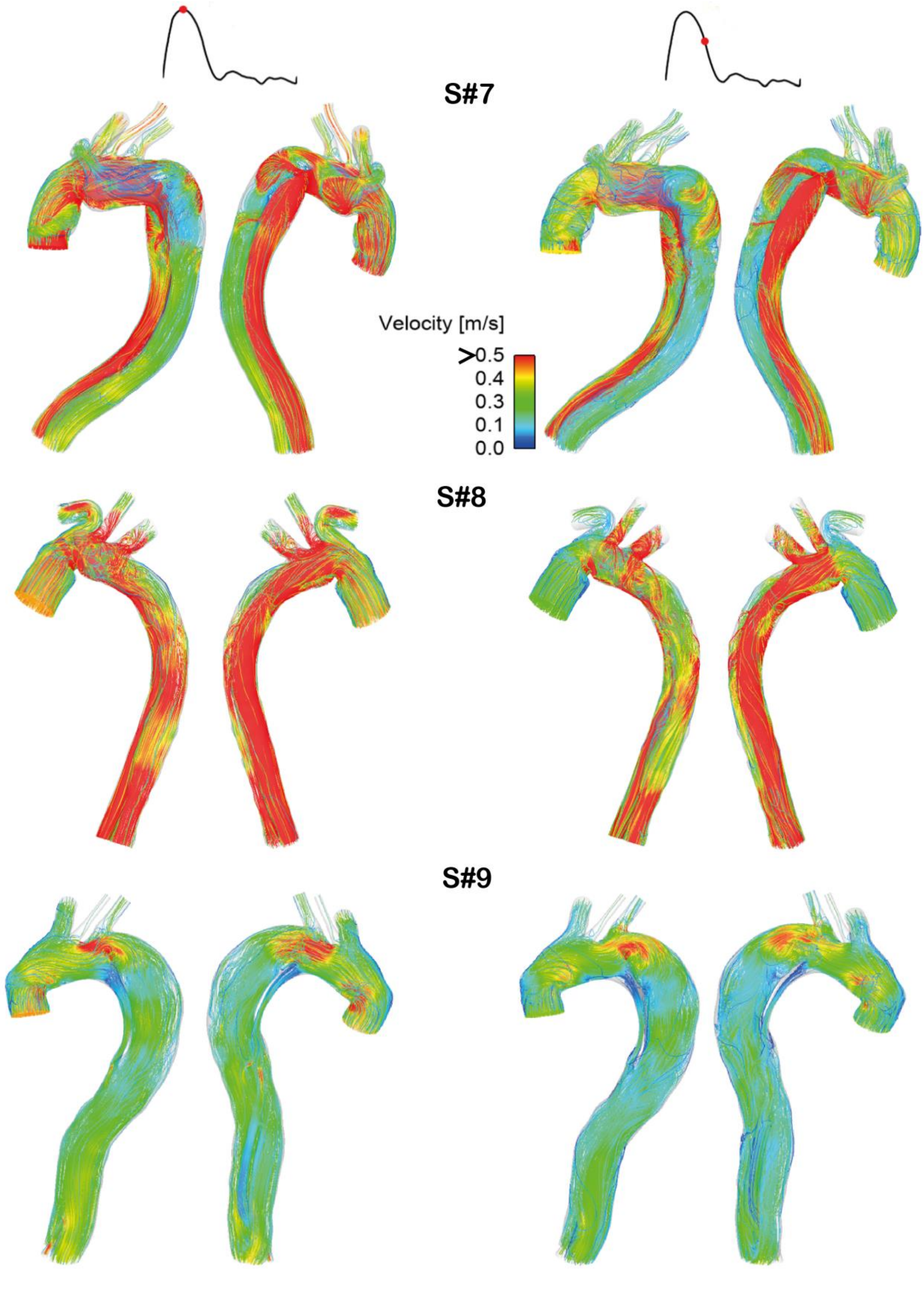
S#7
Velocity [m/s]
>0.5
0.4
0.3
0.1
0.0
S#8
S#9

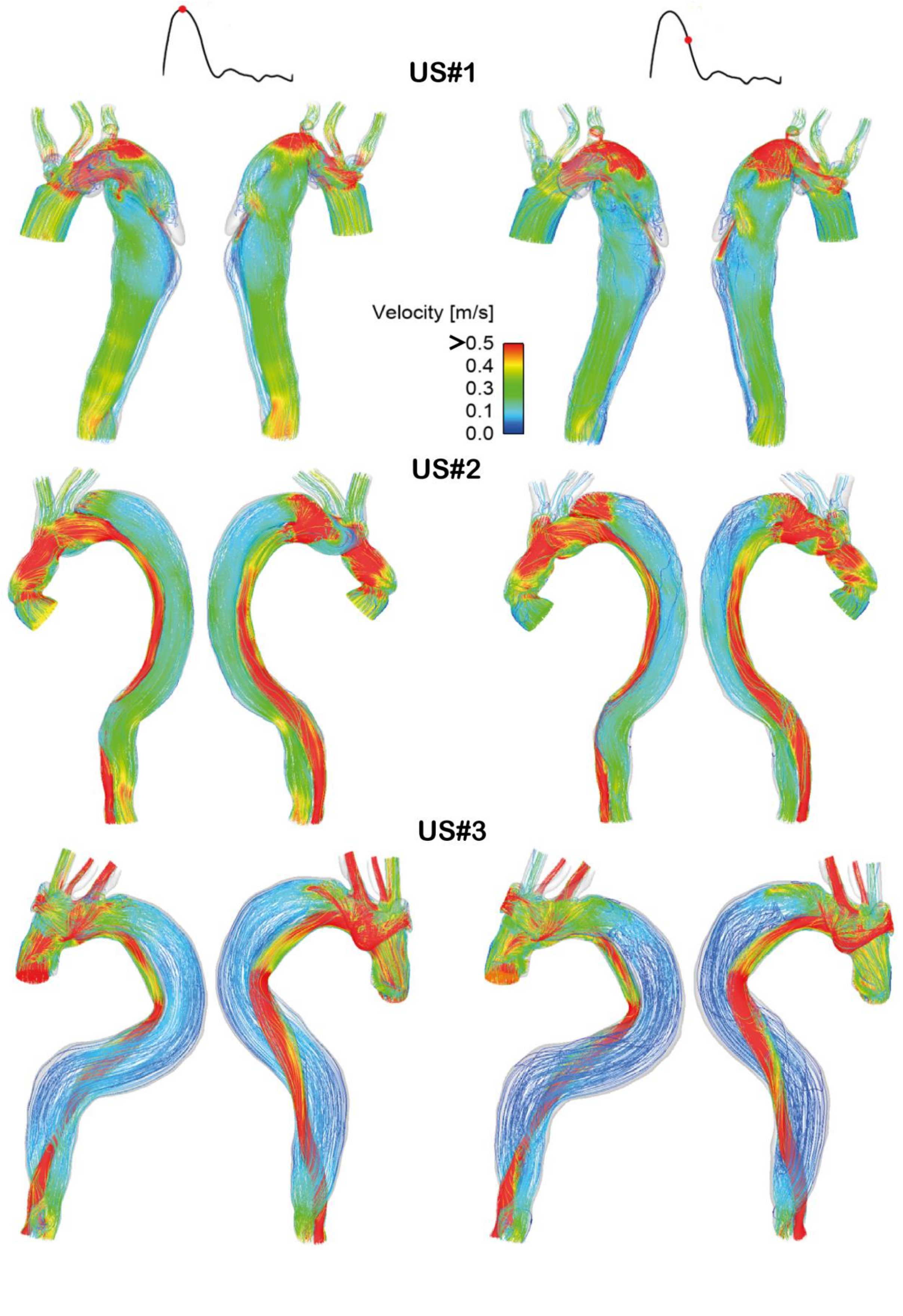

US#1
Velocity [m/s]
>0.5
0.4
0.3
0.1
0.0
US#2
US#3

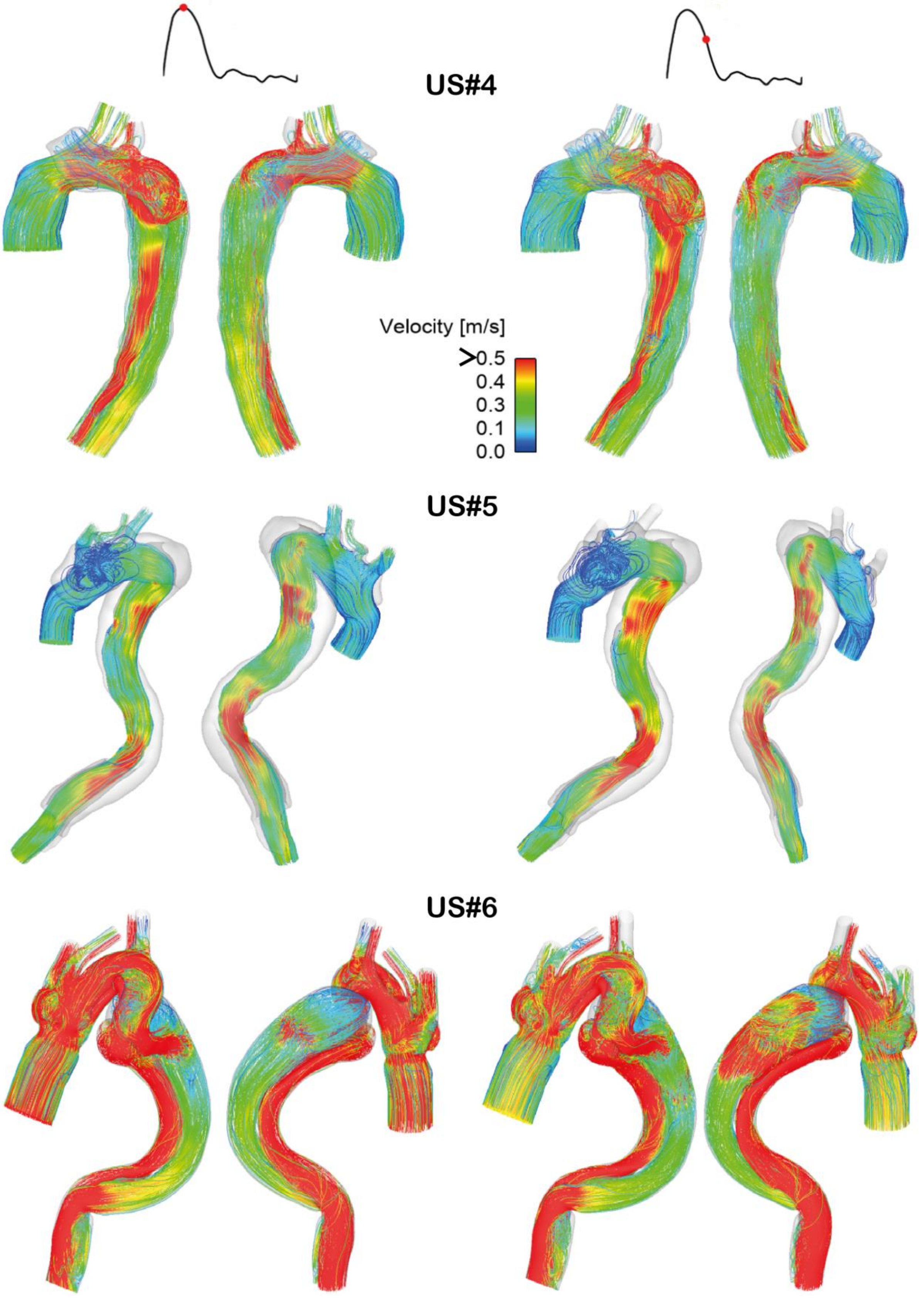
US#4
Velocity [m/s]
>0.5
0.4
0.3
0.1
0.0
US#5
US#6

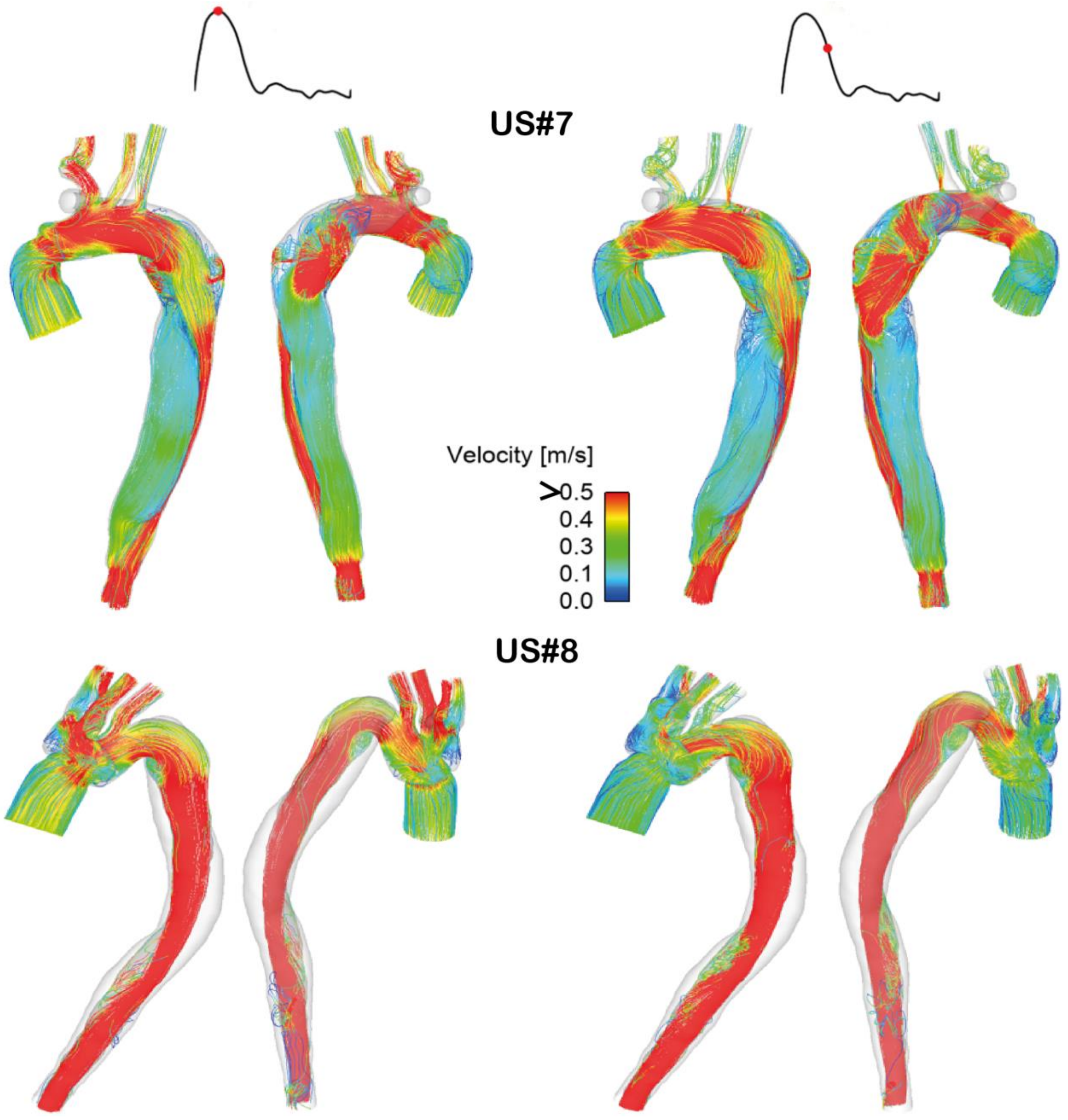

Figure S1. Instantaneous velocity streamlines for all the 17 patient-specific models at two characteristic time points: peak systole (left) and mid-systolic deceleration (right).

S2. Time-Averaged Wall Shear Stress (TAWSS)

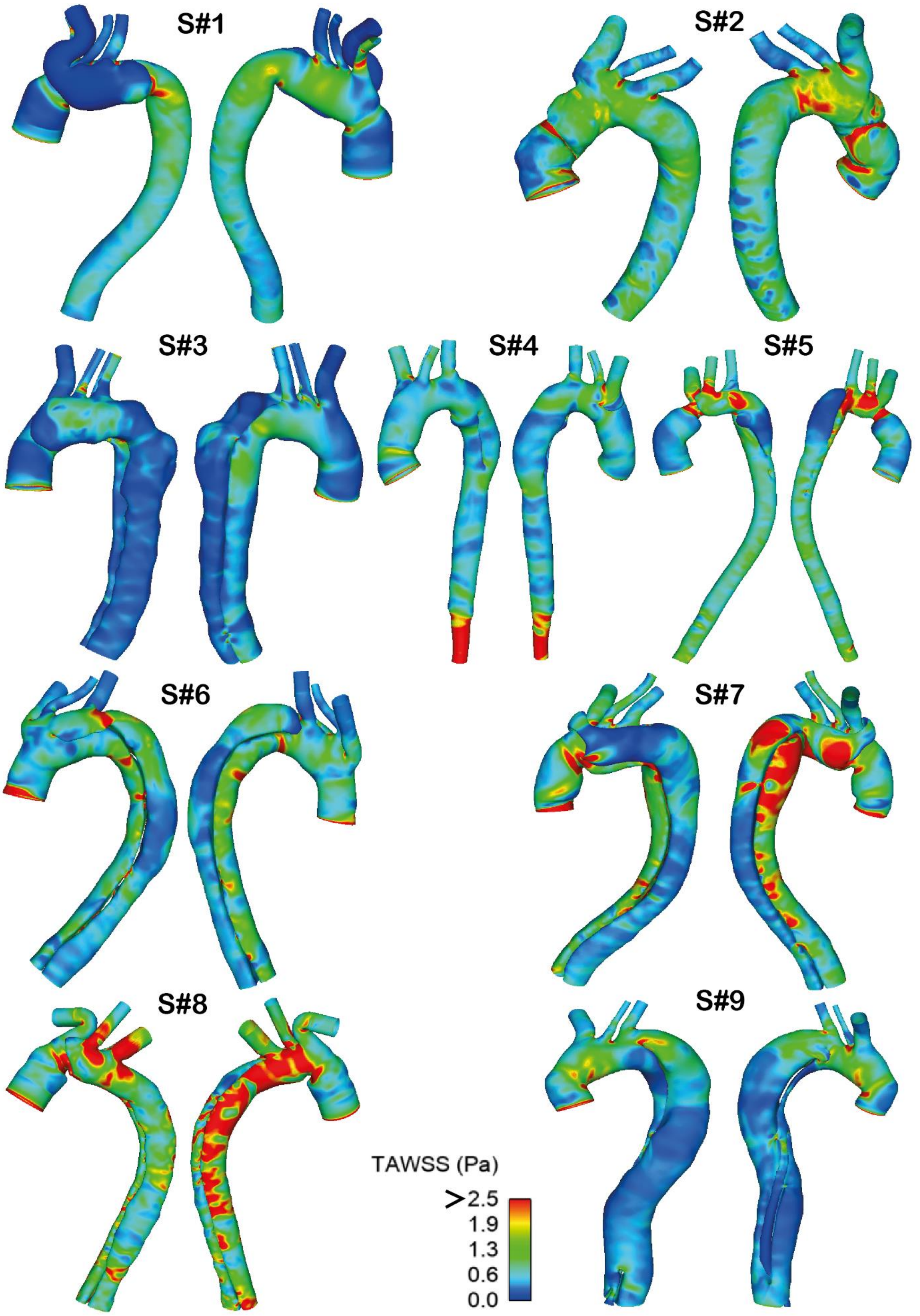

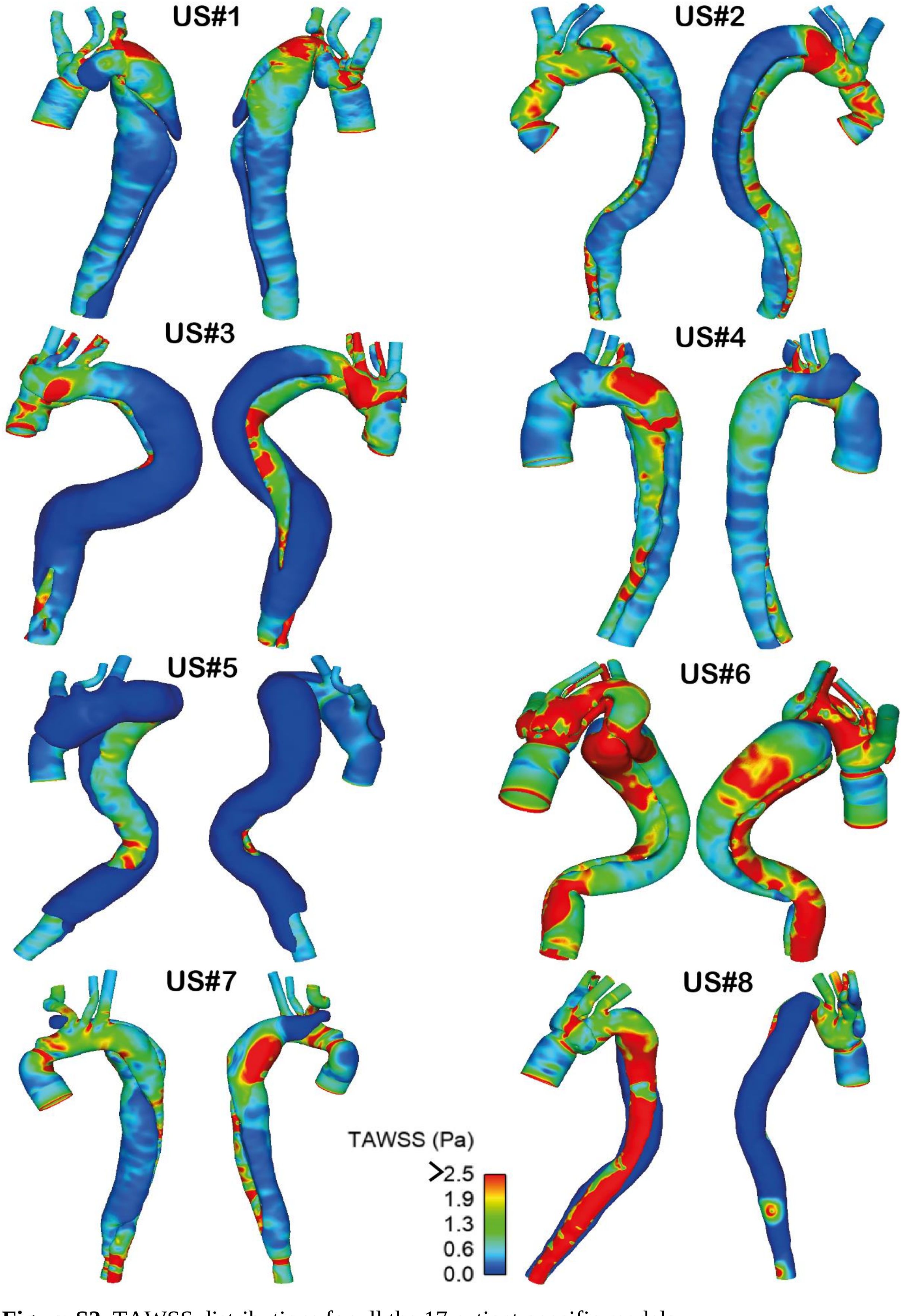

Figure S2. TAWSS distributions for all the 17 patient-specific models.
